# 593. Evaluation of the EDTA-modified Carbapenem Inactivation Method (eCIM) with Using the EDTA Blood Collection Tube for Differentiating the Serine- and Metallo-β-lactamase (MBL) Producing Enterobacterales

**DOI:** 10.1093/ofid/ofad500.661

**Published:** 2023-11-27

**Authors:** Daisuke Sakanashi, Hiroyuki Suematsu, Mina Takayama, Yuzuka Kawamoto, Narimi Miyazaki, Tomoko Ohno, Atsuko Yamada, Akiko Nakamura, Hirotoshi Ohta, Isao Koita, Jun Hirai, Nobuhiro Asai, Nobuaki Mori, Hiroshige Mikamo

**Affiliations:** Aichi Medical University, Nagakute, Aichi, Japan; Aichi Medical University, Nagakute, Aichi, Japan; Aichi Medical University, Nagakute, Aichi, Japan; Aichi Medical University, Nagakute, Aichi, Japan; Aichi Medical University, Nagakute, Aichi, Japan; Aichi Medical University, Nagakute, Aichi, Japan; Aichi Medical University, Nagakute, Aichi, Japan; Aichi Medical University, Nagakute, Aichi, Japan; Aichi Medical University, Nagakute, Aichi, Japan; Aichi Medical University, Nagakute, Aichi, Japan; Aichi Medical University, Nagakute, Aichi, Japan; Aichi Medical University, Nagakute, Aichi, Japan; Aichi Medical University, Nagakute, Aichi, Japan; Aichi Medical University, Nagakute, Aichi, Japan

## Abstract

**Background:**

The rapid and wide spread of carbapenemase-producing Enterobacterales (CPE) has become a global concern. Currently, β-lactams with β-lactamase inhibitors (such as ceftazidime/avibactam, relebactam/imipenem) are used for treatment of CPE infection as one of the effective agents. However, some of combinations do not have activity against MBLs. Thus, we believe that the discriminating between MBL and serine-CPE is important. The eCIM has been published by Clinical & Laboratory Standards Institute (CLSI) to differentiate to MBL from serine-CPE. The eCIM is required EDTA, that is not clinical diagnostic agent. In this study, we examined simplifying the eCIM procedure by using the EDTA blood collection tube (BCT-eCIM).

**Methods:**

A total of 53 CPE (39 MBL and 14 Serin-CPE) were tested. The test population included 39 clinical isolates, and 14 reference strains from NCTC or ATCC **(Table 1)**. The eCIM were performed and interpreted by following CLSI procedures. For BCT-eCIM, whole quantity of 2-mL TSB was inoculated into a BD Vacutainer EDTA Tube (2-mL) (Becton-Dickinson) (Vacutainer-eCIM) or an EDTA-2K Insepack II-D 2-mL tube (Kyokuto Pharmaceutical Industrial, Tokyo, Japan) (Insepack-eCIM); the tubes conform to International Organization for Standardization 6710, hence the 2-mL TSB inoculated tubes obtain final concentration of 4.1 to 6.8 mM EDTA. The BCT-eCIM used these tubes as a substitute for 0.5 mM EDTA TSB tube of eCIM, and any other procedures followed the eCIM procedure. Additionally, we performed the 4 mM- and 7 mM-eCIM to verify the effect of EDTA concentration (variable region of BCT-eCIM).

Table 1

**Figure:**
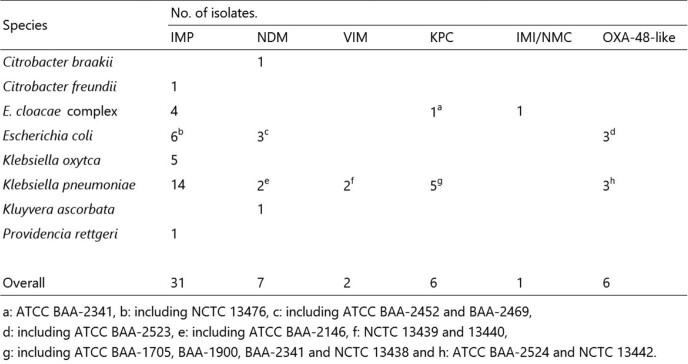

Bacterial strains

**Results:**

**Table 2** and **Figure 1** show the performance data of each eCIMs and the positive and negative Quality control results, respectively. The sensitivity of 4 mM-eCIM, 5 mM-eCIM, 7 mM-eCIM, Vacutainer-eCIM and Insepack-eCIM were 37/40 (93%), 36/40 (90%), 36/40 (90%), 39/40 (98%) and 39/40 (98%), respectively. The specificity of 4 mM-eCIM, 5 mM-eCIM, 7 mM-eCIM, Vacutainer-eCIM and Insepack-eCIM were 11/13 (85%), 13/13 (100%), 13/13 (100%), 12/13 (92%) and 12/13 (92%), respectively.

Table 2

**Figure:**
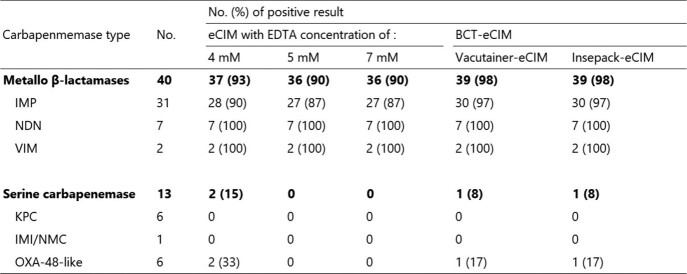

Results obtained using the eCIM and BCT-eCIM

**Figure:**
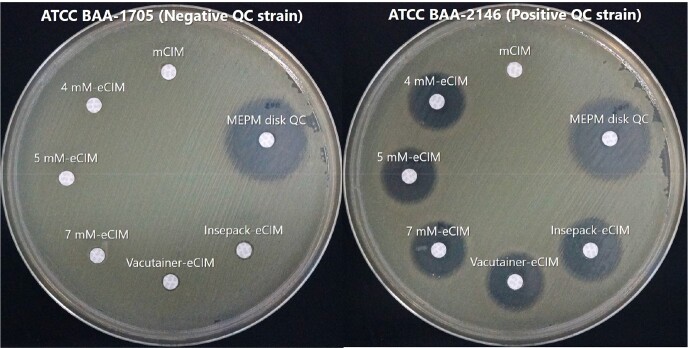

**Conclusion:**

Our methods (BCT-eCIMs) were superior to sensitivity compared to conventional eCIMs. Therefore, BCT-eCIM may be useful in clinical settings as the simplifying method.

**Disclosures:**

**Hiroshige Mikamo, M.D, Ph.D**, Asahi Kasei Pharma Corporation: Grant/Research Support|Merck Sharp & Dohme: Honoraria|Pfizer Inc.: Grant/Research Support|Pfizer R&D Japan: Honoraria|Sumitomo Pharma Co., Ltd.: Grant/Research Support|Sumitomo Pharma Co., Ltd.: Honoraria

